# Early social environment influences the behaviour of a family-living lizard

**DOI:** 10.1098/rsos.161082

**Published:** 2017-05-03

**Authors:** Julia L. Riley, Daniel W. A. Noble, Richard W. Byrne, Martin J. Whiting

**Affiliations:** 1 Department of Biological Sciences, Macquarie University, Sydney, New South Wales, Australia; 2 School of Biological, Earth, and Environmental Sciences, University of New South Wales, Kensington, New South Wales, Australia; 3 School of Psychology and Neuroscience, University of St Andrews, St Andrews, Fife, UK

**Keywords:** asynchronous birth, behavioural flexibility, behavioural syndrome, personality, reptile, social feedback

## Abstract

Early social environment can play a significant role in shaping behavioural development. For instance, in many social mammals and birds, isolation rearing results in individuals that are less exploratory, shyer, less social and more aggressive than individuals raised in groups. Moreover, dynamic aspects of social environments, such as the nature of relationships between individuals, can also impact the trajectory of development. We tested if being raised alone or socially affects behavioural development in the family-living tree skink, *Egernia striolata*. Juveniles were raised in two treatments: alone or in a pair. We assayed exploration, boldness, sociability and aggression repeatedly throughout each juvenile's first year of life, and also assessed social interactions between pairs to determine if juveniles formed dominant–subordinate relationships. We found that male and/or the larger skinks within social pairs were dominant. Developing within this social environment reduced skink growth, and subordinate skinks were more prone to tail loss. Thus, living with a conspecific was costly for *E. striolata*. The predicted negative effects of isolation failed to materialize. Nevertheless, there were significant differences in behavioural traits depending on the social environment (isolated, dominant or subordinate member of a pair). Isolated skinks were more social than subordinate skinks. Subordinate skinks also became more aggressive over time, whereas isolated and dominant skinks showed invariable aggression. Dominant skinks became bolder over time, whereas isolated and subordinate skinks were relatively stable in their boldness. In summary, our study is evidence that isolation rearing does not consistently affect behaviour across all social taxa. Our study also demonstrates that the social environment plays an important role in behavioural development of a family-living lizard.

## 1. Introduction

Conditions during ontogeny affect many aspects of juvenile development. For example, poor nutrition [1] and parasite exposure [2] affect morphological and behavioural development of juveniles and can have long-lasting impacts on individual fitness. The social environment also influences behavioural development, especially in animals with complex social structure. Perhaps the simplest and clearest demonstration of the relationship between social environment and behavioural development comes from experiments that raise social animals in isolation [3,4]. Mammals and birds raised in isolation show decreased activity, exploration, boldness and sociability, but increased fear and aggression[5–8]. These effects on behaviour can be long-lasting, and significantly reduce individual fitness because of abnormal behaviour [5,8]. However, the relationship between social environment and development has predominately been studied in overtly social taxa, specifically mammals and birds. Little is known about the developmental impacts of social environment in less-studied social taxa, like insects, fishes, amphibians, and reptiles.

The limited literature examining how social environment impacts reptile development indicates trends similar to studies on birds and mammals. For example, hatchling veiled chameleons (*Chamaeleo calyptratus*) raised in isolation are more submissive during social interactions and had lower performance in a foraging task [9], which suggests decreases in sociability and boldness. Similarly, water snakes (*Natrix maura*) that were incubated alone were less social than snakes that were incubated in contact with other eggs [10]. Such similarity between studies across social taxa suggests isolation rearing may consistently affect behavioural development across species. However, none of these studies on reptilian behaviour examined the temporal stability of effects produced by social isolation, which limits our understanding of whether such effects are long-lasting or are variable over time. It is also important to realize that the impact of social environment on development is not always as simple as just the presence/absence of conspecifics [11,12]. A social environment is dynamic, and interactions between all individuals within a social group contribute to an individual's experience and development.

Dominant–subordinate relationships often form between individuals in social groups [13]. An individual's social rank, and the social interactions it experiences as a result of this rank, can affect its behaviour and fitness [14–16]. Dominance hierarchies, which are often size-based, can form among juveniles if they are raised together, and especially if that litter or clutch is born or hatched asynchronously. Asynchronous birth or hatching describes the time span between the birth of the first and last offspring within a litter or clutch [17], and is widespread across many animals (e.g. insects [18], sharks [19], amphibians [20], reptiles [21], and birds [15]). An asynchronously produced juvenile's social rank has been found to affect their individual growth rate, survival, and behaviour [15,16]. For example, the order of hatching in some nidicolous birds leads to siblicide of younger hatchlings by older clutch mates [22]. If social experience due to differences in social rank affects juvenile development, then the resulting divergence in sibling phenotype and behaviour may promote variable life-history strategies that could ensure survival of at least one offspring if faced with an unpredictable environment.

We examined whether social environment affects behavioural development in a social lizard, the tree skink (*Egernia striolata*). Females have asynchronously born litters ranging in size from one to four juveniles ([23], JL Riley 2015, unpublished data), and wild populations aggregate in social groups made up of either mating adult pairs, parents with offspring, or just juveniles [24,25]. In the laboratory, we reared juveniles in isolation or with another conspecific (a social environment). We examined the dynamics within the social environment to determine if pairs were forming dominant–subordinate relationships. Also, we quantified four behavioural traits (exploration, boldness, sociability, and aggression; table 1) of juveniles across their first year of life. We asked four questions:

(1) Are tree skink behavioural traits correlated?
(2) Does social environment impact individual phenotype?
(3) Does social environment affect behavioural development?
  a. We predicted that isolation would impact behavioural development similarly to how it has in other social taxa; isolated skinks would be less social, exploratory, and bold, as well as more aggressive compared with socially reared lizards.
  b. We predicted evidence of a dominant–subordinate relationship between socially reared pairs, and also expected that these social interactions would influence individual phenotype and behaviour.
(4) Does social environment influence temporal consistency of behavioural traits?

**Table 1.** Behavioural traits we quantified and their descriptions within the context of our study. We also detail how the behavioural traits were measured, and what the resulting response variable was for our statistical analyses.

behavioural trait	description	quantification	behavioural variable
exploration	— how a skink assesses and searches a novel environment — varies from sedentary to exploratory	— from videos we scored: (1) time spent moving within a novel environment (s) (2) number of times entered refuges within a novel environment — conducted a PCA with the two variables to combine them into an exploratory score	— exploratory score (PC1) — as the value increases, the skinks are more exploratory
boldness	— how a skink reacts to a risky situation — varies from brave (risky) to shy (safe)	— scored the latency to return to basking (s) after a ‘predator’ chase	— latency to return to bask (s) — as the value increases, skinks are less bold
sociability	— how a skink aggregates with conspecifics — varies from solitary (avoidance) to social (aggregative)	— scored how far away (mm) the focal skink was from a conspecific every 10 min during the 5 h assay — then, calculated the weighted average distance each focal skink was from the conspecific across the whole trial	— sociality score — lower values reflect more aggregative behaviour, and higher values reflect avoidance
aggression	— how a skink reacts in a defensive scenario — varies from a fight (aggressive) to flight (passive) response	— from an interaction with a replica, scored: (1) number of mouth gapes (2) number of bites (3) latency to retreat (s) — conducted a PCA with the three variables to combine them into an aggression score	— aggression score (PC1) — lower values indicate a higher aggressive response from the skink (i.e. more bites, more gapes, longer latency to retreat)

## 2. Material and methods

We collected gravid tree skinks from Albury, New South Wales (35.98′ S, 146.97′ E) in January 2014 and 2015, and monitored their parturition at Macquarie University. In our study, tree skink litter size ranged from one to four juveniles (mean = 2.25, 95% CI: 2.04 to 2.47). Tree skinks give birth to a litter, within captivity, over a mean of 1.6 days (95% CI: 1.45 to 1.75, *N* = 55; JL Riley 2015, unpublished data) and each juvenile within a litter was born within a mean of 0.77 days (95% CI: 0.70 to 0.84; JL Riley 2015, unpublished data) of each other. We sampled 28 juveniles in 2014 and 38 juveniles in 2015 (see the electronic supplementary materials for more details on our study species, captive husbandry, and measurements recorded).

### 2.1. Behavioural trait assays

After all juveniles within a cohort were born, we carried out an initial ‘baseline’ assay. We then randomly allocated juveniles into two treatments: isolated and social. The isolated treatment housed lizards alone (*N*_2014_ = 14 lizards, *N*_2015_ = 16 lizards), while the social treatment housed two unrelated juveniles together (*N*_2014_ = 14 lizards within 7 pairs, *N*_2015_ = 22 lizards within 11 pairs). Both treatments were representative of wild social rearing environments because juveniles have been observed both alone and in pairs ([24,25], JL Riley 2016, unpublished data), although most commonly, social groups consist of parent(s) and one to three offspring [23]. Including parents in our social treatment was not feasible because adult *Egernia* spp. can be highly aggressive towards juveniles [26,27] and infanticide can occur [26,28,29]. Once within their treatments, we assayed behavioural traits three more times: around five months of age, seven months of age and 12 months of age (see the electronic supplementary material for exact dates).

Each assay period was 12 days in duration, and consisted of eight ‘trait assays’ and four ‘break days’ (electronic supplementary material, figure S1). Owing to the size of our experimental room, we had to measure juvenile behaviour within two batches (maximum of 24 per batch; see the electronic supplementary materials for details on batch allocation and sample sizes). During assays, skinks were individually housed in rectangular arenas (690 mm W × 470 mm L × 455 mm H), and assays were remotely video recorded (see the electronic supplementary materials for details). Each assay aimed to categorize one of four behavioural traits: exploration, boldness, sociability, and aggression (table 1).

### 2.2. Exploration assays

To categorize exploratory behaviour, we introduced skinks into a novel environment (e.g. akin to an open-field test). We repeated this procedure on two subsequent days (electronic supplementary material, figure S1). The first arena had a sand substrate, two black refuge boxes (120 mm W × 175 mm L × 38 mm H) with one entrance on the inner side placed at each end of the arena, and four white clay tiles (47 mm W × 47 mm L × 4 mm H) placed in the middle of the arena (electronic supplementary material, figure S2). The second arena had a grid-paper substrate and two black refuge boxes (same dimensions and orientation as above; electronic supplementary material, figure S2). At the beginning of each trial, we introduced the lizard into the arena within a central, containment refuge. The skink acclimated within this refuge for 5 min. The trial started when we lifted the central refuge, and then the trial ran for 30 min (electronic supplementary material, video S1).

From video recordings, we scored: (i) time spent moving (s) and (ii) number of times they entered a refuge. These two measures were combined using a principal component analysis (PCA). Our PCA used the correlation matrix because our variables were on different scales and this approach standardizes the data [30]. The PCA was performed using the *princomp* function in R v 3.0.3 [31]. These two behaviours were highly correlated and positively loaded on a single component (electronic supplementary material, table S1). We used the first principal component (PC1) in further analyses as our ‘exploratory score’; as the value increases, it reflects more exploratory behaviour (table 1).

### 2.3. Boldness assays

We categorized skink boldness with three predator-response assays that occurred on consecutive days (electronic supplementary material, figure S1). For our boldness assays, we positioned the heat lamp directly over one of the lateral refuges, which resulted in a ‘hot’ refuge (electronic supplementary material, figure S2). We placed an ice pack beneath the other refuge, under the arena, resulting in a ‘cold’ refuge with temperatures outside the skink's optimal range (electronic supplementary material, figure S2). After set-up, we left the skink for 1 h to ensure it was either on top of, or within the ‘hot’ refuge. Then, we simulated a predatory attack by ‘chasing’ the skink away with a gloved hand from the ‘hot’ refuge until it entered the ‘cold’ refuge at the opposite end of the arena [32]. We then recorded the skink's behaviour for 45 min after it entered the ‘cold’ refuge (electronic supplementary material, video S1). We scored the time (s) it took the skink to return to the ‘hot’ refuge. If the skink did not return, we assigned it a value of 2700 s. We used the latency to return to the ‘hot’ refuge as our quantification of boldness; lower values indicate bolder behaviour (table 1).

### 2.4. Social assays

Our social assay began by placing the skink into the central refuge. We then inserted a clear, Perspex® divider 110 mm from the front of the arena (electronic supplementary material, figure S2). An unrelated female was randomly assigned to each juvenile, and placed on the opposite side of the Perspex to that of the juvenile. The refuge was lifted, and we recorded the juvenile and female for 5 h (electronic supplementary material, video S1).

From the video, at 10 min intervals over 5 h, the juvenile was scored as being in one of four lateral quadrats (110 mm width) in relation to the female (electronic supplementary material, figure S2). We calculated the weighted average distance the juvenile was from the conspecific across the entire trial by multiplying the number of times in each quadrat (Q1, Q2, Q3 and Q4) by the average distance the quadrat was away from the female (55 mm, 110 mm, 165 mm and 220 mm, respectively) and dividing the product by the number of observations (*N*_obs_ = 30). The distance the juvenile was located from the conspecific during the trial was used as a ‘sociality score’ in further analyses; lower values were reflective of the juvenile being closer to the adult conspecific (table 1).

### 2.5. Aggression assays

Our aggression assay was based on the methods outlined by Sinn *et al*. [27] and was repeated on two consecutive days (electronic supplementary material, figure S1). The arena was set up with the heat lamp pointed at one end of the bin, and the refuge at the opposite side (electronic supplementary material, figure S2). After set-up, we allowed 1 h for the skink to begin to bask beneath the heat lamp. The aggression assay began when a researcher approached the bin, and then waited within view of the skink for 30 s. After this, the researcher touched the nose of the basking skink with a soft Plasticine replica of a juvenile tree skink attached to the end of a stick. The skink was tapped on the nose every 12 s over a maximum of 2 min (i.e. maximum of 10 taps). The assay ended once the skink retreated into the refuge (electronic supplementary material, video S1). The replica skink was size-matched to the average snout–vent length (SVL) and total length of experimental skinks at each assay time period (i.e. the replica grew in size at the same rate as the juveniles).

We scored: (i) number of times a skink gaped and/or (ii) bit the replica, and (iii) the time (s) until the skink took refuge within the hide. If the skink did not take refuge within the hide, it was assigned a time of 120 s. These three measures were combined using a PCA using their correlation matrix [30]. These behaviours were highly intercorrelated and all negatively loaded on a single component (electronic supplementary material, table S1). We used the first principal component (PC1) in further analyses as our ‘aggression score’; lower values indicate greater aggression (table 1).

### 2.6. Social dynamics within socially reared pairs

We quantified social dynamics between socially reared pairs to determine if they were forming dominant–subordinate relationships. After all behavioural assays were completed for the 2014 cohort, we observed the social pair interacting during reintroduction into their mutual captive housing tub (for housing details, see the electronic supplementary materials) after being separated for 18 days for a learning experiment [33]. We observed skinks after this period of separation (18 days) because we assumed they would have to re-establish their dominance–subordinate relationship upon reintroduction. We recorded the social pair interacting for 15 min [25]. Before observations, we marked each individual with numbered cloth tape (Tesa®, Germany) to allow identification. From recordings, we counted the following behaviours for each individual:

(1) ‘Bite’: the focal skink grasps another with its jaws {3}
(2) ‘Nip’*:* the focal skink grasps another with its jaws and then releases immediately, less than 1 s later {3}
(3) ‘Chase’: the focal skink follows another fleeing skink {2}
(4) ‘Lie-on’*:* the focal skink lies on another such that some part of its head or body is on top of the other skink {1}
(5) ‘Move-over’: the focal skink moves over the top of another skink {1}
(6) ‘Flee’: the focal skink moves away from another chasing skink {−2}
(7) ‘Move-away’: the focal skink moves away from another skink {−1}

To quantify the role each individual played in the interaction, we multiplied the number of times each of the behaviours occurred by its weight (in curly brackets above), and summed to create an aggression score [9,26]. Weights for behavioural scores were guided by Ballen *et al*. [9] and Langkilde *et al*. [34]. Submissive behaviours (6 and 7) had negative weights, so they reduced the aggression score of the individual.

We compared aggression scores of social pairs to determine if individuals were dominant or subordinate. Six of seven social pairs differed in their aggression score: all individuals that were categorized as dominant (higher aggression score) were larger (SVL) than the subordinate (lower aggression score) individual (two sample *t*-test; *t*_12_ = 2.55, *p* = 0.03). Of these pairs, three of six contained both males and females, and all of the males were categorized as ‘dominant’. As these trends were consistent across pairs, we categorized all socially reared skinks as dominant if they had a larger SVL and/or were the only male within their social pair. Herein, we refer to individuals from the social environment simply as ‘dominant’ or ‘subordinate’.

### 2.7. Statistical analyses

Prior to conducting analyses, we explored the data following Zurr *et al*. [35] (see the electronic supplementary material). We used a Bayesian analysis framework with a Markov chain Monte Carlo (MCMC) sampling approach to fit all our models. To fit our models (see specific details below), MCMC was applied using the *MCMCglmm* R package [36]. We used a burn-in of 150 000 iterations, a thinning rate of 1350, and 1 500 000 iterations for each posterior distribution. Uniform priors were used for the regression, and inverse Wishart priors for variance parameters (*V* = 1, *nu* = 0.002 [36]). Continuous covariates in all models were mean-centred prior to analysis. We visually inspected all trace plots from our models to ensure chains were well mixed. Autocorrelation of the chains of both fixed and random effects was assessed to ensure levels were low (lag < 0.1) using the *autocorr* function, and we also performed Geweke and Heidelberg autocorrelation diagnostics (from the R package *coda* [37]). Model assumptions of normality of residuals and homogeneity of variance were verified ([35]; see the electronic supplementary materials). In our results, we present pooled posterior modes and 95% credible intervals. Parameter estimates were considered significant when the credible intervals did not include 0, and the *pMCMC* values calculated by *MCMCglmm* were less than 0.05 [36]. When we predicted fitted lines from the models for visualizations, we set the continuous fixed factors to the mean and the factorial fixed factors to the intercept-level values.

#### 2.7.1. Are behavioural traits correlated?

To evaluate whether there were correlations between our four behavioural traits, we used a multi-response linear mixed-effects model [38,39]. Our model included random intercepts for each behavioural trait, and a random intercept and slope for juvenile identity across age (details in the electronic supplementary materials about model variable selection). We assumed a multivariate normal distribution with a variance–covariance structure specifying the within- and between-individual variance and covariances among behaviours [36,38]. We used a temporally comparable subset of data for this model: data from the first replication of a behavioural trait (i.e. explore A, bold A and aggression A; see the electronic supplementary material, figure S1) at 5, 7 and 12 months of age (*N*_obs_ = 153, *N*_juv_ = 51).

#### 2.7.2. Does social experience impact individual phenotype?

We compared the number of lizards that partially or fully autotomized their tails at least once with a Fischer's exact test. Then, we examined whether skink (i) SVL (mm) and (ii) relative tail length (RTL = tail length/SVL) differed among isolated, dominant and subordinate skinks. RTL was used as a proxy for the frequency of tail autotomy experienced by each individual—we expected that tail length decreases in relation to the number of times it is autotomized [40]. We fitted identical Gaussian linear mixed-effects models (LMMs) for each response variable. Fixed factors were *social experience* (categorical with three levels), *age* (days; continuous), *cohort* (categorical with two levels), and an *interaction between social experience and age*. To incorporate the dependency among observations of the same individual, we included a random intercept and slope for juvenile identity across age; and to incorporate the dependency among observations of lizards from the same litter, we included a random intercept for mother identity. We also included a random intercept for housing tub to incorporate dependency among observations of lizards from the same captive environment. The model with RTL as the response variable only included data from the 2015 cohort. We calculated contrasts between dominant and subordinate skinks by comparing posterior distributions from model estimates.

#### 2.7.3. Does social experience affect behavioural development?

We separately performed four identical Gaussian LMMs for each behavioural trait to assess how they were affected by skink *social experience* (categorical with three levels). Models also included the fixed factors of *age* (continuous), *batch* (categorical with two levels), *cohort* (categorical with two levels), *body temperature* (continuous), and *sex* (categorical with two levels) [39,41]. We also included a random intercept and slope for juvenile identity across age, and random intercepts for mother identity and housing tub. We also included an *interaction between social experience and age*, but if the interaction was not significant, we removed and refitted the model. Our boldness variable (latency to return to bask, s) was log-transformed to normalize the data, and we used a rank transformation to normalize our aggression score [42]. Also, for the model with aggression as the response variable, we included data from only time periods 1–3 (electronic supplementary material, figure S1).

#### 2.7.4. Does social experience influence consistency of behavioural traits?

Repeatability, in general, refers to the extent to which individual differences in traits are maintained over time [38,41,43], and, in the case of this study, quantifies consistency of our four behavioural traits. To calculate repeatability for each behavioural trait, we calculated adjusted repeatability from the above models examining how behavioural traits differed between social experiences (i.e. *R|time* from Biro and Stamps [41], but in our case the time variable was skink age). For comparison, we also modelled each behavioural trait with LMMs that only contained an intercept and the random factors of juvenile identity, mother identity and housing tub (electronic supplementary material, table S2). From these models, we calculated LMM-based repeatability that did not consider additional explanatory factors (adapted *R*_*M*_ [38,39,43]). Finally, we subset the data by social experience and reran the original models (fixed and random factors varied as appropriate depending on the subset of data; electronic supplementary material, tables S3–S5). We then calculated adjusted repeatability from those models [41], and compared it between social experience qualitatively.

## 3. Results

### 3.1. Are behavioural traits correlated?

**Figure 1.**
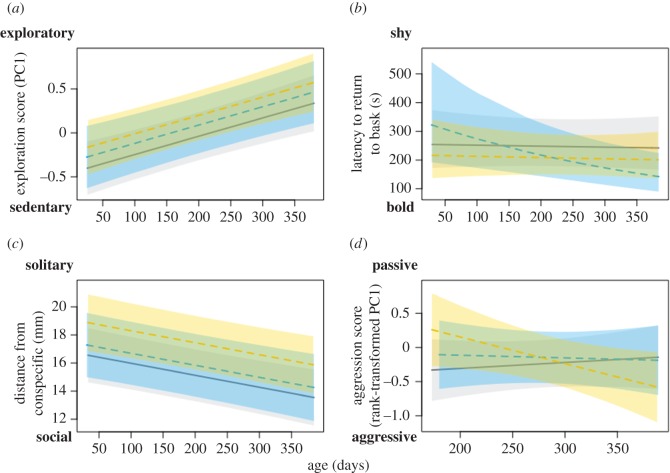
Tree skink social experience (isolated: grey polygon with solid line, dominant: blue polygon with dashed line, and subordinate: yellow polygon with dashed line) affects behavioural traits differently. (*a*) Exploration did not differ between social experiences. (*b*) Dominant skinks increased in their boldness (i.e. the inverse of latency; table 1) as they aged, whereas isolated and subordinate skinks maintained their level of boldness over time. (*c*) Subordinate skinks were less sociable than isolated skinks, but there was no difference in sociability between subordinate and dominant skinks, nor dominant and isolated skinks. (*d*) Subordinate skinks increased in aggression (i.e. the inverse of our aggression score; table 1) as they aged, and this change in aggression was different from that of isolated skinks but not different from that of dominant skinks. Aggression was also not different between dominant and isolated skinks. Shaded polygons around predicted fitted means are 95% predicted credible intervals.

Aggression and exploration were significantly negatively correlated (*ρ*_exploration, aggression_ = −0.668, 95% CI = −1.000 to −0.068) at the between-individual level; which means that tree skinks that had a higher exploration score (representing more explorative behaviours; see figure 1*a*) would have a lower aggression score (representing more aggressive behaviours; see figure 1*d*). All other between- and within-individual covariances and correlations were not significant (table 2).

**Table 2.** Variance (dark grey shaded), covariance (white background) and correlation (light grey shaded) estimates between the four behavioural traits for (*a*) between- and (*b*) within-individual error with associated 95% credibility intervals (in brackets) testing for correlation between behavioural traits in *Egernia striolata*. We bolded variables if the 95% credible intervals did not include 0 to indicate their significance.

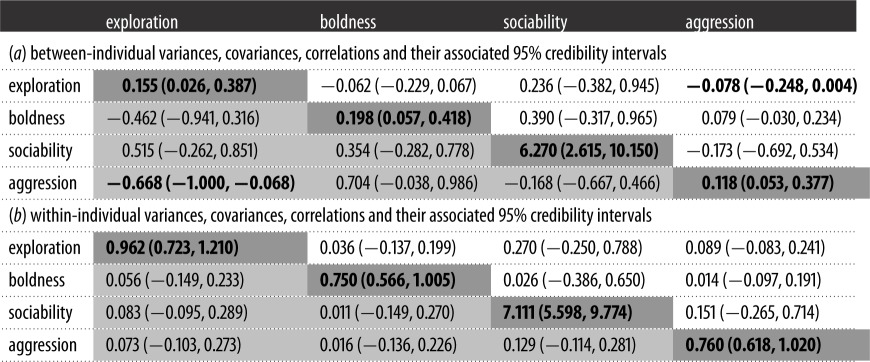

### 3.2. Does social experience impact individual phenotype?

**Figure 2.**
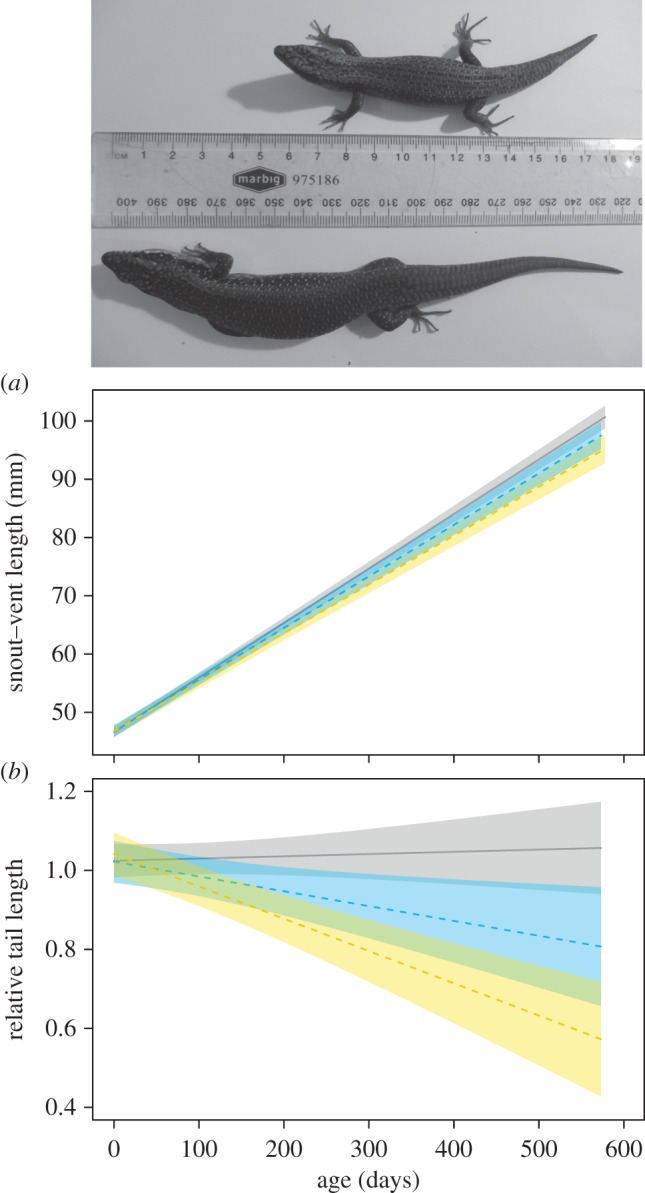
(*a*) Subordinate tree skinks (yellow polygon and dashed line) had smaller snout–vent lengths than isolated skinks. Isolated skinks (grey polygon and solid line) increased in SVL faster than dominant (blue polygon and dashed line) and subordinate skinks. (*b*) RTL, as well as the rate of change in RTL, differed between all social experiences (photograph shows difference in size found within a social pair). Shaded polygons around predicted probabilities are 95% predicted credible intervals.

Isolated skinks were significantly larger in SVL than subordinate skinks (table 3; figure 2*a*). SVL did not differ between isolated and dominant skinks, or subordinate and dominant skinks (*β*_DomSub_ = 0.966, 95% CI = −0.369 to 2.547; table 3 and figure 2*a*). Isolated skink growth rate was significantly faster than subordinate skinks, and marginally faster (*pMCMC* = 0.086) than dominant skinks (table 3 and figure 2*a*). Growth rate was not different between dominant and subordinate skinks (*β*_DomSub_ = 0.727, 95% CI = −0.180 to 1.819).

**Table 3.** Outcomes of linear mixed-effects models testing if SVL and RTL were affected by age (mean-centred), social experience (isolated, ISO; dominant, DOM; and subordinate, SUB), and the interaction between age × social experience. Cohort (2014 and 2015) was also included in the model examining SVL, but not the model examining RTL (as indicated with ‘n/a’). The models also included the random effects of juvenile identity, mother identity, and housing tub.

	SVL (mm)				RTL			
	*N*_obs_ = 903, *N*_juv_ = 66, *N*_mom_ = 35, *N*_tub_ = 48				*N*_obs_ = 432, *N*_juv_ = 37, *N*_mom_ = 20, *N*_tub_ = 27			
fixed effects	*β*	2.5%	97.5%	*pMCMC*	*β*	2.5%	97.5%	*pMCMC*
intercept (ISO and 2014)	69.868	68.892	70.954	<0.001^a^	1.053	0.986	1.086	<0.001^a^
age	15.813	15.086	16.284	<0.001^a^	0.004	−0.029	0.046	0.580
social experience: DOM	−1.138	−2.473	0.411	0.138	−0.091	−0.170	−0.018	0.008^a^
social experience: SUB	−2.295	−3.700	−0.846	0.004^a^	−0.187	−0.251	−0.101	<0.001^a^
cohort	1.964	0.800	2.593	0.002^a^	n/a	n/a	n/a	n/a
age × social experience: DOM	−0.767	−1.718	0.110	0.086^b^	−0.078	−0.135	−0.013	0.020^a^
age × social experience: SUB	−1.506	−2.526	−0.741	0.002^a^	−0.169	−0.212	−0.092	<0.001^a^

random effects	*σ^2^*	2.5%	97.5%		*σ^2^*	2.5%	97.5%	
juvenile identity	11.224	7.488	17.727		0.019	0.010	0.036	
mother identity	0.003	0.000	0.947		0.001	0.000	0.004	
housing tub	0.003	0.000	0.978		0.001	0.000	0.003	
residual	12.206	10.640	12.991		0.011	0.010	0.013	

^a^Indicates significant variables.

^b^Indicated marginally significant variables.

Fifty-five percent (6/11) of dominant skinks and 73% (8/11) of subordinate skinks partially or fully autotomized their tails at least once. By contrast, no isolated skinks (0/16) autotomized their tails (Fisher's exact test, *p* < 0.001). The rate of change in RTL over time was significantly different among all social environments (isolated, dominant, and subordinate), which means RTL of isolated skinks grew at almost an equivalent rate to their SVL, but tails of subordinate skinks grew the slowest probably because of multiple instances of caudal autotomy (*β*_DomSub_ = 0.067, 95% CI = 0.009 to 0.144; table 3 and figure 2*b*).

### 3.3. Does social experience affect behavioural development?

Skink exploration did not differ among social experience (*β*_DomSub_ = −0.154, 95% CI = −0.434 to 0.204), nor did it change differently among social experiences as skinks aged (table 4 and figure 1*a*).

**Table 4.** Outcomes of linear mixed-effects models examining if behavioural traits of *Egernia striolata* differed among social experiences (ISO, isolated; DOM, dominant; SUB, subordinate). These models also included the fixed factors of age, batch (1 or 2), cohort (2014 and 2015), body temperature, and sex (male or female), as well as the random effects of juvenile identity, mother identity, and housing tub. All continuous variables were mean-centred. The social experience × age interaction effect was included if it was significant, but if it was removed from the model due to non-significance then it is indicated with an ‘n/a’.

	exploration				boldness				sociability				aggression			
	*N*_obs_ = 496, *N*_juv_ = 62, *N*_mom_ = 35, *N*_tub_ = 46				*N*_obs_ = 672, *N*_juv_ = 56, *N*_mom_ = 31, *N*_tub_ = 44				*N*_obs_ = 244, *N*_juv_ = 61, *N*_mom_ = 31, *N*_tub_ = 45				*N*_obs_ = 300, *N*_juv_ = 50, *N*_mom_ = 30, *N*_tub_ = 39			
fixed effects	*β*	2.5%	97.5%	*pMCMC*	*β*	2.5%	97.5%	*pMCMC*	*β*	2.5%	97.5%	*pMCMC*	*β*	2.5%	97.5%	*pMCMC*
intercept (ISO, 1, FEMALE, and 2014)	−0.001	−0.281	0.270	0.916	5.537	5.201	5.843	<0.001^a^	15.004	13.028	16.716	<0.001^a^	−0.188	−0.548	0.158	0.178
age	0.206	0.133	0.317	<0.001^a^	−0.026	−0.136	0.102	0.776	−0.883	−1.338	−0.508	<0.001^a^	0.093	−0.105	0.249	0.542
social experience: DOM	0.141	−0.133	0.387	0.346	−0.259	−0.509	0.120	0.218	0.681	−0.889	2.614	0.430	0.060	−0.220	0.427	0.616
social experience: SUB	0.302	−0.071	0.548	0.138	−0.138	−0.484	0.173	0.298	2.086	0.440	4.157	0.016^a^	0.131	−0.379	0.393	0.842
batch: 2	0.241	0.082	0.449	0.012^a^	−0.212	−0.357	−0.054	0.004^a^	−0.661	−1.501	0.106	0.102	−0.190	−0.466	0.038	0.096
cohort: 2015	−0.459	−0.743	−0.187	<0.001^a^	0.596	0.237	0.898	<0.001^a^	−0.777	−2.624	1.068	0.516	0.530	0.147	0.811	0.010^a^
body temperature	0.192	0.091	0.272	<0.001^a^	−0.131	−0.198	−0.054	<0.001^a^	0.267	−0.145	0.633	0.282	0.071	−0.036	0.211	0.196
sex: MALE	0.246	−0.100	0.468	0.162	−0.014	−0.308	0.293	0.972	1.364	−0.676	3.021	0.270	0.105	−0.282	0.427	0.588
age × social experience: DOM	n/a	n/a	n/a	n/a	−0.250	−0.409	−0.013	0.022^a^	n/a	n/a	n/a	n/a	−0.084	−0.364	0.175	0.536
age × social experience: SUB	n/a	n/a	n/a	n/a	−0.053	−0.201	0.193	0.942	n/a	n/a	n/a	n/a	−0.245	−0.598	−0.029	0.034^a^

random effects	*σ^2^*	2.5%	97.5%		*σ^2^*	2.5%	97.5%		*σ^2^*	2.5%	97.5%		*σ^2^*	2.5%	97.5%	
juvenile identity	0.005	0.001	0.245		0.003	0.001	0.148		0.078	0.003	9.226		0.005	0.001	0.225	
mother identity	0.001	0.000	0.098		0.002	0.000	0.121		0.042	0.000	8.619		0.001	0.000	0.078	
housing tub	0.002	0.000	0.109		0.131	0.001	0.231		0.014	0.000	5.126		0.002	0.000	0.119	
residual	0.907	0.777	1.006		0.820	0.709	0.893		7.688	5.954	10.311		0.819	0.665	0.968	

repeatability	*R*	2.5%	97.5%		*R*	2.5%	97.5%		*R*	2.5%	97.5%		*R*	2.5%	97.5%	
*R*_*M*_ from intercept-only model	0.002	0.000	0.056		0.001	0.000	0.082		0.001	0.000	0.451		0.003	0.001	0.170	
*R*_*ADJ*_|*age* from full model	0.013	0.002	0.206		0.003	0.001	0.134		0.004	0.000	0.505		0.047	0.001	0.244	

^a^Indicates significant variables.

Boldness did not differ among social experiences (*β*_DomSub_ = −0.093, 95% CI = −0.337 to 0.263). However, as dominant skinks aged, they became bolder. This was significantly different from isolated skinks that were stable in their boldness over ontogeny, and marginally different from subordinate skinks that were also stable in their boldness (*β*_DomSub_ = −0.238, 95% CI = −0.440 to 0.009; table 4 and figure 1*b*).

Subordinate skinks were less social than isolated skinks, but did not differ in sociability from dominant skinks (*β*_DomSub_ = −1.348, 95% CI = −3.877 to 0.434; table 4). Isolated and dominant lizards did not differ in their sociability (table 4 and figure 1*c*). Sociability did not change differently among social experiences as skinks aged (table 4).

Aggression did not differ among social experiences (*β*_DomSub_ = 0.112, 95% CI = −0.342 to 0.429; table 4). However, subordinate skink aggression increased with age, and this was significantly different from isolated skink aggression, which slightly decreased as they aged (table 4). Change in aggression over ontogeny did not significantly differ between dominant and subordinate, or dominant and isolated, skinks (*β*_DomSub_ = 0.156, 95% CI = −0.062 to 0.537; table 4 and figure 1*d*).

### 3.4. Does social experience influence consistency of behavioural traits?

**Figure 3.**
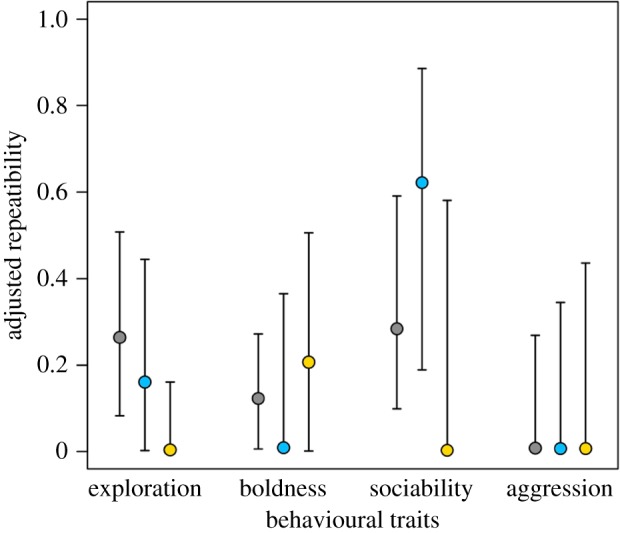
Adjusted repeatability (*R*_*ADJ*_|*age*) varies among social experiences of tree skinks (isolated, grey circle; dominant, blue circle; subordinate, yellow circle) depending on the behavioural trait. *R*_*ADJ*_|*age* of exploration and boldness was low for all social experiences, and *R*_*ADJ*_|*age* of aggression was no different from 0 for all social experiences. By contrast, *R*_*ADJ*_|*age* of sociability was moderate for dominant skinks, low for subordinate skinks, and no different from 0 for isolated skinks. Differences between social experiences are not significant because of the large overlap of 95% credibility intervals.

Repeatability, calculated from intercept-only models (*R*_*M*_; electronic supplementary material, table S2), was lower than the adjusted repeatability calculated from full models (*R*_*ADJ*_*|age*) that also considered covariates (table 4). *R*_*ADJ*_*|age* for exploration, boldness, aggression and sociability scores was low (table 4). Between social experiences *R*_*ADJ*_*|age* varied from low (e.g. subordinate skink exploration *R*_*ADJ*_*|age* = 0.004, 95% CI = 0.001, 0.161) to high (e.g. dominant skink sociability *R*_*ADJ*_*|age* = 0.622, 95% CI = 0.189, 0.886), although, due to the overlap of 95% credibility intervals, there was no significant differences in *R*_*ADJ*_*|age* among social experiences (figure 3).

## 4. Discussion

### 4.1. Are behavioural traits correlated?

Juvenile tree skinks that were more explorative than others also tended to be more aggressive towards a size-matched conspecific, regardless of social experience. These were the only two of the four behavioural traits we quantified that were correlated. Correlation between behavioural traits interests behavioural ecologists because it may suggest that correlated traits are selected upon simultaneously and may not be free to evolve independently [39,44]. In animal personality research, a correlation between two or more behavioural traits across multiple observations is termed a ‘behavioural syndrome’ [45,46]. Our findings agree with the bulk of current findings about correlations between behavioural traits, which typically show that more exploratory individuals are also more aggressive (e.g. shy–bold, reactive–proactive continuum of traits [45,47–49]). We currently do not know much about the ecology and life history of tree skink juveniles in the wild, and thus how covariance of these two traits may benefit an individual is unknown. However, in a closely related skink, White's skink (*Liopholis whitii*), juveniles’ dispersal from their natal group is thought to vary depending on the degree of relatedness to their social father [50]. Juveniles that are from extra-pair matings, and are thus not related to the male within the social group, have a larger home range that only overlaps with their mother and siblings [50]. So, in the case of these more dispersive and more exploratory juveniles, it may pay to be more aggressive in encounters with size-matched siblings to gain resources like maternal access, food, and shelter space that are more limited to them. Alternatively, the correlation may simply be explained by this species’ ecology. Tree skinks live in a constrained habitat (i.e. crevices), and within this habitat the likelihood of encountering conspecifics is high. Perhaps tree skinks that are more exploratory inhabit a larger home range, and thus are also more aggressive, so they can defend their territory from other conspecifics. Overall, more research is needed to understand the fitness benefits of trait covariance in this social lizard species.

### 4.2. Does social experience impact individual phenotype?

Individual social experience across ontogeny impacted morphological development in tree skinks. Our socially reared pairs formed dominant–subordinate relationships wherein skinks that were larger and/or male were consistently dominant in their pairings. While we standardized resources between social and isolated treatments, growth rate of socially reared skinks was slower than in isolated skinks. This may have been the result of resource competition (e.g. for food or basking locations, or increased activity and energy waste from social interactions) between pairs that was a product of the dominant–subordinate relationship, as has been shown in other lizard species. For example, Stamps [51] found that juvenile bronze anoles (*Anolis aeneus*) with high territory overlap experienced reduced growth rate due to increased food competition. The hypothesis that socially reared pairs were competitive and aggressive towards each other is also supported by the fact that the majority (73%) of subordinate skinks autotomized their tails at least once, and the RTL of subordinate skinks decreased sharply over time instead of remaining stable. Tail loss is also a consequence of juvenile aggression in other *Egernia* spp. (e.g. the black rock skink, *E. saxatilis* [26]), suggesting that the social environment may be more costly for juveniles than previously thought. There are substantial costs of tail autotomy (e.g. reduced motor ability, energetic costs, indirect effects on communication, reduced survival and fecundity [40,52]), and impacts are more extreme for juveniles than adults because they need to allocate energy towards both tail regeneration and somatic growth [40]. The physical impacts on development we observed within the social treatment indicate that the social environment is costly, especially for subordinate (smaller in size and/or female) skinks.

### 4.3. Does social experience affect behavioural development?

Contrary to our prediction, isolation rearing did not affect behavioural development in ways expected from the literature. We expected that isolated skinks would be less exploratory, less bold, less social and more aggressive than socially reared conspecifics. Instead, isolated skinks were more social than subordinate skinks, opposite to our prediction. Also, isolated skinks remained consistently bold and aggressive, whereas we predicted a change in both these traits. Thus, being raised in isolation did affect behavioural development in tree skinks, yet not in the way previously observed in other taxa [3,9,53–55]. In most mammals and birds, isolation rearing greatly hinders individual development and fitness. Although we did not measure fitness consequences of the behavioural differences we found for isolated tree skinks, the behavioural changes we observed (higher sociability) may not have significant negative impacts on individual fitness. This finding has important implications for reptile conservation and management projects, as well as ethical considerations. It is important to consider each target animal's sociality in any research and management project; however, our findings suggest that isolation rearing does not consistently, negatively impact behaviour across all social taxa. In wild populations of tree skinks and other *Egernia* group species, developing in isolation is an option [24,25]: for example, in White's skink growing up in isolation and/or with reduced social contact often occurs for a proportion of offspring in each litter (e.g. juveniles unrelated to the social father; [56]). Since variable social exposure occurs over development in these social lizards, they may be predisposed to cope well with isolation rearing. From this hypothesis, we would predict that a mammal or bird with a similar social structure and ecological constraints as the tree skink would show the same, unexpected, reaction to social isolation.

As expected, with dominant–subordinate relationships within socially raised pairs, behavioural development differed between these social experiences. Dominant skinks increased in boldness as they aged, which was significantly different from isolated skinks and marginally different from subordinate skinks that remained stable in their boldness over time. Perhaps, as they repeatedly ‘won’ in social interactions and/or recognized they were the larger skink within their environment, they were more inclined to exhibit risky behaviour [42]. Subordinate skinks were the least social and increased in aggression over time, yet these trends were only significantly different from isolated skinks, not from dominant skinks. This lack of significance, despite a clear trend, was probably due to large variation in skink behaviour. After aggressive encounters with their social partner, subordinate skinks may have tended to avoid social interactions and learnt to react aggressively to conspecifics over time. Our experiment differs from natural conditions because social groups lacked the presence of parent(s) [23] and lizards were restricted within their housing tubs; however, this restriction of space may not be so different from natural conditions because tree skink social groups are always constrained by restricted resources (i.e. crevices and hollows [23,57]). Our findings demonstrate that behavioural development is affected by social feedback experienced during the juvenile life stage. This may help explain why asynchronous birth that generates social rivalries within litters, a common phenomenon across multiple taxa, might be an adaptive strategy.

Many *Egernia* group species, including tree skinks, give birth over several days or weeks[21,58–60]. Research on White's skink has shown that embryo development prior to birth is synchronous and that females retain offspring to delay births over time [21], suggesting this may be an adaptive behaviour. Within litters that are born asynchronously, a size-based dominance hierarchy forms, and the youngest and smallest offspring exhibit reduced growth rates and increased mortality because of competition and aggression from siblings [15,16,22]. In our study, the difference in age between social pairs (range from 1 to 18 days) is an analogous comparison to juveniles within an asynchronously born litter. We examined if the difference in age between social pairs was related to the difference in their aggression scores, and found that social pairs that were more different in age tended to also exhibit a larger difference in aggression (see the electronic supplementary material, figure S3). This relationship was not significant, probably because of our limited sample size, but the trend suggests that competitive asymmetries are more pronounced in juvenile social pairings that are more different in age. Thus, in our study, older lizards had a head start on growth and were able to more easily dominate younger individuals, which is exactly what occurs in litters born asynchronously [16,17,22]. We found that subordinate lizards were less social and became more aggressive over time. These behavioural traits might be linked to a higher rate of dispersal away from social groups. Our evidence that social rank within a social group impacts development provides support for the hypothesis that asynchronous birth may be beneficial because it promotes divergence in offspring traits, and potentially life-history strategies, which could ensure survival of at least one offspring if faced with an unpredictable environment. These insights from our laboratory experiment provide promising avenues for future research directly testing the adaptive value of asynchronous birth of *Egernia* group species in the wild.

### 4.4. Does social experience influence consistency of behavioural traits?

Our adjusted repeatability scores were very low for all four behavioural traits. Also consistency in behavioural traits did not differ between lizards within different social environments, and was highly variable between individuals. Thus, tree skink behavioural traits were not temporally consistent over their first year of life, nor can they be classified as ‘personality’ traits as they can be referred to in animal behaviour [46,61]. Contrasting with our results, in a study that assayed adult tree skink behavioural traits twice over 12 days, there was high to medium repeatability in exploration, boldness and aggression, but no repeatability in sociability and activity [39]. There is a disparity in the scale of time that behavioural traits were examined between these two studies (behaviour is more likely to be consistent over 12 days than 1 year), but perhaps juveniles undergoing development are generally more variable in behaviour because they are more sensitive to change than during adulthood. Currently, there are only a few studies that examine the development of personality traits, yet they consistently have demonstrated that personality (or behavioural) traits are less temporally stable in juveniles when compared with adults [62–64]; our findings corroborate this trend.

## 5. Conclusion

In summary, we found evidence that the social environment (isolation, dominant member of a pair and subordinate member of a pair) impacts behavioural development in tree skinks. To the best of our knowledge, this is the first time social environment has been shown to play an important role in the behavioural development of a reptile, an under-studied taxon in regard to social behaviour [65]. Many environmental factors (e.g. predation pressure, incubation temperature, nutrition; [62,66–68]) impact behavioural development and cause behavioural divergence. Our study highlights that both lack of social interaction and the nature of social interactions are other factors that can potentially drive change and divergence across behavioural development. Our study also helps to inform an often-overlooked area of personality research: how behavioural traits may change over the course of development. Specifically, our results corroborate previous work that found that juvenile personality is not temporally stable.

Overall, our study provides evidence that isolation rearing does not consistently impact behaviour across all social taxa. Tree skinks cope well in isolation rearing, perhaps due to existing social variation within wild rearing conditions. Other social species, with a similarly varied social structure, may also exhibit this coping ability for isolation rearing. A more thorough understanding of behavioural development across all social taxa might also be gained from our evidence that suggests that social rank within a group of conspecifics could influence behavioural divergence in an adaptive manner when growing up in a variable environment.

## Supplementary Material

Supplementary Material

## Data accessibility

All data and R code for analyses used in this study can be accessed from the Bitbucket repository: https://julia_riley@bitbucket.org/julia_riley/e-striolata-personality.git.
