# Supplementary material for: Early social environment influences the behaviour of a family-living lizard
Source: R Soc Open Sci. 2017 May 3;4(5):161082. doi: 10.1098/rsos.161082 (PMC5451802; doi:10.1098/rsos.161082)
Supplement: Supplementary Material [file rsos161082supp1.pdf]

Early social environment influences the behaviour of a family-living lizard

Julia L. Riley, Daniel W.A. Noble, Richard W. Byrne, and Martin J. Whiting
Corresponding author: Julia L. Riley, Macquarie University, julia.riley87@gmail.com

Supplementary Materials

Materials and methods

Study species

Tree skinks (*Egernia striolata*) are a medium-sized, viviparous skink found across southeastern Australia [1]. This skink typically resides within cracks or hollows within trees and rocks [1] and exhibits variable social organization both within and between populations. Within a population, individuals can either be found alone or in groups of variable size (2-10 skinks; [2,3]). Across the tree skink's range, different social systems have been described between populations. In some arboreal populations, tree skinks have been found in small groups (maximum of 3 individuals) and are often found alone [2,4]. In other arboreal and in saxicolous populations, tree skinks were often observed in large social groups (< 10 individuals) of closely related individuals [3,5-8].

Field collection and captive housing

Gravid female tree skinks were collected from Albury, New South Wales (35.98'S, 146.97'E) in January 2014 (N = 15) and 2015 (N = 20), and maintained at Macquarie University until parturition. Parturition occurred in 2014 from 10 February to 12 March, and in 2015 from 17 January to 10 February. The females were kept in a climate-controlled room (maintained at 24°C) within opaque plastic tubs (350 mm W x 487 mm L x 260 mm H). These tubs were lined with newspaper, and contained tree bark, a water dish, and a refuge (120 mm W x 175 mm L x 38 mm H). Each skink's tub was lit by a UV lamp, and had under-cage heating wire limited to one side of the tub to allow thermoregulation. All housing tubs were cleaned once weekly. Each skink was fed 3 adult crickets dusted with calcium and vitamins twice a week, and puréed fruit (1.25 ml of Heinz® baby food: apple and mango, apple, and pear) once weekly. Gravid females were fed individually within their housing tubs.

During parturition, we checked the females twice a day to see if they had given birth. Offspring were separated from females immediately post-parturition (within 12h), and

housed separately until all females had given birth. Before separation, we measured each juvenile's snout-vent length (mm, SVL: the distance between the tip of the snout and the posterior edge of the cloaca), total length (mm), and tail length (mm) with a standard ruler to the nearest 1 mm. We also recorded mass with a digital scale (SP6001, Scout Pro, Ohaus, Pine Brooks NJ, USA) to the nearest 0.01 g, and marked each juvenile with a unique toe-clip [9]. We re-measured each skink monthly throughout their first year of life and at a maximum of a week prior to each behavioural assay. During each measurement period, we also noted if skinks had complete or damaged tails (i.e., were their tails autotomized). We calculated relative tail length (RTL = tail length/SVL), and used this in our analyses as a proxy for the frequency of tail autotomy in each individual; if a skink underwent multiple instances of tail autotomy their RTL would be lower and would decrease over time instead of remaining stable (around 1 [10]). The offspring were housed within smaller opaque plastic tubs than the females (85 mm W x140 mm L x 60 mm H). All other housing criteria were the same, except they were fed nymph crickets instead of adult crickets. Skinks were fed within their housing tubs; so socially-reared skinks were fed in the presence of their pair. In 2014, a total of 28 juveniles were included in our experiments, and in 2015 a total of 38 juveniles were sampled. During winter months (June to August), skinks were maintained at a reduced temperature (18°C).

Timing and Sample Sizes of Behavioural Assays

Baseline behavioural assays occurred from 23 March to 14 April 2014, and 15 April to 9 May 2015. We repeated the behavioural assays three more times within each year: (1) ca. 5 months of age (23 August to 18 September 2014, and 19 September to 7 October 2015), (2) ca. 7 months of age (29 October to 21 November 2014, and 3 to 26 November 2015), and (3) ca. 12 months of age (31 January to 23 February 2015, and 18 January to 10 February 2016).

Due to the size of our experimental room, we had to measure juvenile behaviour within two batches (maximum of 24 per batch). Juveniles were randomly allocated to a batch for each assay period, and there were approximately equal numbers of skinks within each batch. In the 2014 cohort (N =28), 12 skinks were in batch 1 and 16 skinks were in batch 2 during the baseline assays. At 5 and 7 months of age, 18 and 10 skinks were in batch 1 and 2, respectively. At 12 months of age, 15 and 13 skinks were in batch 1 and 2, respectively. In the 2015 cohort (N = 38), 19 skinks were in both batch 1 and 2 during the baseline assays. At

5 months of age, 22 and 16 skinks were in batch 1 and 2, respectively. At 7 and 12 months of age, 18 and 20 skinks were in batch 1 and 2, respectively.

Experimental housing and data collection

During behavioural assays, all skinks were housed in rectangular, opaque, plastic arenas (690 mm W x 470 mm L x 455 mm H) within a climate-controlled room. Between behavioural assays, we cleaned arenas thoroughly with F10SC® (F10 Products Limited, Loughborough, UK) to remove olfactory cues. Each arena was warmed on one side with a heat lamp, and contained a refuge and a water dish except during assays. Skinks were fed as usual (see above) but only after assay completion each day. Immediately after assays finished each day, body temperature of each skink was measured using an infrared thermometer (accuracy of 1.5°C, model # RIT310, Ryobi, Techtronic Industries Australia Pty Ltd, Doncaster, Victoria). We recorded behavioural assays using a mounted security camera system (CCTV security systems, Melbourne, Victoria), and behaviour was scored from these videos. Videos were scored blind to social treatment, and all videos were scored by one person (JLR) to avoid inter-observer bias.

Additional information about statistical analyses

Initially, in our univariate LMMs to assess differences in behavioural traits, we included *body condition index* (residuals from a simple linear regression between log-transformed mass and SVL) as a covariate, but it was not significant in any model. So, we removed it in order to implement the simplest model with the most power in our analyses.

In the multi-response LMM to assess correlations between all four behavioural traits, we did consider if maternal effects and housing tub effects should also be included in the model. However, we opted to use the simplest model, with only juvenile identity, to ensure the interpretation of within- and between-individual behavioural trait variance, covariance, and correlations were clear.

Results

Data exploration and model validation

During data exploration for all models, we did not find any unexplainable outliers, and no strong collinearity was found between predictor variables. We did remove missing values

where applicable, thus there are a variable number of observations and individuals within each model (sample sizes are provided in all tables).

Mixing of our chains was good in the two models that examined the response variables of SVL and RTL, as well as all four models that examined behavioural traits. Visual inspection of residual plots for each model did not reveal any obvious deviations from homoscedasticity or normality of residuals.

Relationships between covariates and behavioural traits

The fixed effects (age, batch, cohort, body temperature, and sex) each differently influenced behavioural traits (table 4). Sex was not significantly related to any behavioural trait. Individuals spent more time exploring as they aged, exploration was positively related to body temperature, skinks from our second batch were more exploratory, and skinks from the 2015 cohort were less exploratory (table 4). Skinks increased in boldness (a lower latency to return to bask) as they increased in body temperature, skinks from the second batch were bolder, and skink from the 2015 cohort were less bold (table 4). As skinks aged they situated themselves, on average, closer to conspecific adults (higher sociability; table 4). Skinks from our 2015 cohort were less aggressive than skinks from our 2014 cohort (table 4).

Relationship between difference in age and aggression score between social pairs

We examined if absolute difference in aggression score was related to the absolute difference in age (days) between social pairs using a simple linear regression (function *lm* from the *base* R package [11]). We found that as age difference increased, so did the difference in aggression score ($t_{1,5} = 1.914$, $P = 0.114$, $R^2 = 0.308$; Fig. S3). However, this relationship was not significant probably because of our small sample size (N = 7).

References

1. Cogger HG. 2014 *Reptiles and amphibians of Australia*. 7 edn. Clayton, VIC: CSIRO Publishing.

2. Bustard HR. 1970 A population study of the scincid lizard Egernia striolata in northern New South Wales. *KNAW* **73**, 186-213.

3. Bonnett MP. 1999 *The ecology, behaviour and genetic relationships of a population of Egernia striolata*. Honours Thesis. Adelaide, SA: Flinders University of South Australia.

4. Cunningham RB, Lindenmayer DB, Crane M, Michael D, MacGregor C. 2007 Reptile and arboreal marsupial response to replanted vegetation in agricultural landscapes. *Ecol Appl* **17**, 609–619. (doi:10.1890/05-1892)

5. Swanson S. 1976 *Lizards of Australia*. Sydney, NSW: Angus and Robertson.

6. Ehmann H. 1992 *Encyclopedia of Australian Animals. Reptiles.* Sydney, NSW: Angus and Robertson.

7. Michael DR, Cunningham RB. 2010 The social elite: habitat heterogeneity, complexity and quality in granite inselbergs influence patterns of aggregation in *Egernia striolata* (Lygosominae: Scincidae). *Austral Ecol* **35**, 862–870. (doi:10.1111/j.1442-9993.2009.02092.x)

8. Duckett PE, Morgan MH, Stow AJ. 2012 Tree-dwelling populations of the skink *Egernia striolata* aggregate in groups of close kin. *Copeia* **2012**, 130–134. (doi:10.1643/ce-10-183)

9. Borges-Landaez, PA, Shine R. 2003 Influence of toe-clipping on running speed in *Eulamprus quoyii*, an Australian Scincid lizard. *J Herp* **37**, 592–595. (doi:10.1670/26-02N)

10. Bateman PW, Fleming PA. 2009 To cut a long tail short: a review of lizard caudal autotomy studies carried out over the last 20 years. *J Zool* **277**, 1–14. (doi:10.1111/j.1469-7998.2008.00484.x)

11. R Core Team. 2016 *A language and environment for statistical computing*. Vienna, Austria: R Foundation for Statistical Computing.

12. Nakagawa S, Schielzeth H. 2010 Repeatability for Gaussian and non-Gaussian data: a practical guide for biologists. *Biol Rev Camb Philos Soc*. **85**, 935–956. (doi:10.1111/j.1469-185X.2010.00141.x)

13. Biro PA, Stamps JA. 2015 Using repeatability to study physiological and behavioural traits: ignore time-related change at your peril. *Anim Behav* **105**, 223–230. (doi:10.1016/j.anbehav.2015.04.008)

Supplemental Videos

Video S1. Behavioural assay procedures. Videos are speed up 2x to increase the speed of tree skink behaviours for viewing, and edited to highlight the important behaviours scored in our study.

Supplemental Tables

Table S1. Component loadings from our principal component analysis (PCA) for the behaviours scored from our *E. striolata* exploration and aggression assays. The PCA for the exploration assay consisted of two behaviours (time spent moving in a novel environment, and number of times a skink entered two refuges within the new environment). The PCA for the aggression assay consisted of three behaviours (latency to retreat into a refuge after being repeatedly touched by a replica skink, number of times the skink gaped, and number of times it bit the replica). These traits were measured for 66 skinks (within two cohorts; $N_{2014} = 28$, $N_{2015} = 38$) eight times (two times during four time-periods within each skinks' first year of life; $N_{obs} = 792$).

**Behaviour**	**Component one loadings**
Exploration	
Time spent moving (s)	0.707
Number of times entered refuges	0.707
Aggression	
Number of gapes	-0.429
Number of bites	-0.657
Latency to retreat into the refuge (s)	-0.651

Table S2. Outcomes of intercept-only linear mixed-effects models used for estimating repeatability (adapted R_M from Nakagawa and Schielzeth [12]) of behavioural traits in *Egernia striolata*. The model also included the random effects of juvenile identity, mother identity, and housing tub. Significant parameters in each model are indicated asterix (*) to the right of the 95% credibility intervals and the *pMCMC* values.

	Exploration N_{obs} = 496, N_{juv} = 62, N_{mom} = 35, N_{tub} = 46				
**Fixed effects**	***β***	**2.5%**	**97.5%**	***pMCMC***	
Intercept	0.024	-0.130	0.152	0.886	
**Random effects**	***σ²***	**2.5%**	**97.5%**		
Juvenile identity	0.002	0.000	0.068		
Mother identity	0.002	0.000	0.155		
Housing tub	0.002	0.000	0.162		
Residual	1.019	0.908	1.186		
**Repeatability**	***R***	**2.5%**	**97.5%**		
R_M	0.002	0.000	0.056		

	Boldness N_{obs} = 672, N_{juv} = 56, N_{mom} = 31, N_{tub} = 44				
**Fixed effects**					
Intercept	5.679	5.481	5.840	<0.001	*
**Random effects**					
Juvenile identity	0.001	0.000	0.092		
Mother identity	0.003	0.000	0.232		
Housing tub	0.121	0.000	0.290		
Residual	0.854	0.773	0.958		
**Repeatability**					
R_M	0.001	0.000	0.082		

	Sociability N_{obs} = 244, N_{juv} = 61, N_{mom} = 31, N_{tub} = 45				
**Fixed effects**					
Intercept	15.477	14.639	16.166	<0.001	*
**Random effects**					
Juvenile identity	0.034	0.000	8.079		
Mother identity	0.017	0.000	5.075		
Housing tub	0.005	0.000	5.075		
Residual	9.769	8.136	12.203		
**Repeatability**					
R_M	0.001	0.000	0.451		

	Aggression N_{obs} = 300, N_{juv} = 50, N_{mom} = 30, N_{tub} = 39			
**Fixed effects**				
Intercept	-0.000	-0.157	0.150	0.956
**Random effects**				
Juvenile identity	0.003	0.000	0.166	
Mother identity	0.002	0.000	0.088	
Housing tub	0.002	0.000	0.170	
Residual	0.833	0.676	0.959	
**Repeatability**				
R_M	0.003	0.001	0.170	

Table S3. Results of linear mixed-effects models that only examined data for isolated skinks used for estimating repeatability (adapted $R|time$ from Biro and Stamps [13]) of behavioural traits. These models also include the fixed factors of age, batch (1 or 2), cohort (2014 and 2015), body temperature, and sex (male or female), as well as the random effect of juvenile identity. We highlighted important variables in each model with an asterix (*) to the right of the 95% credibility intervals and the *pMCMC* values.

	Exploration N_{obs} = 224, N_{juv} = 28, N_{mom} = 28				
**Fixed effects**	**β**	**2.5%**	**97.5%**	***pMCMC***	
Intercept	0.019	-0.263	0.411	0.790	
Age	0.259	0.101	0.385	0.002	*
Batch (ref = 1)	0.331	-0.002	0.555	0.034	*
Cohort (ref = 2014)	-0.445	-0.924	-0.132	0.024	*
Body temperature	0.118	0.041	0.305	0.012	*
Sex (ref = FEMALE)	0.030	-0.289	0.389	0.582	
**Random effects**	**σ^2**	**2.5%**	**97.5%**		
Juvenile identity	0.281	0.020	0.647		
Residual	0.755	0.651	0.961		
**Repeatability**	***R***	**2.5%**	**97.5%**		
$R_{ADJ}|age$	0.264	0.083	0.508		

	Boldness N_{obs} = 312, N_{juv} = 26, N_{mom} = 26				
**Fixed effects**					
Intercept	5.601	5.262	5.991	<0.001	*
Age	0.005	-0.130	0.119	0.894	
Batch (ref = 1)	-0.281	-0.497	-0.015	0.030	*
Cohort (ref = 2014)	0.428	0.087	0.846	0.028	*
Body temperature	-0.108	-0.212	-0.008	0.040	*
Sex (ref = FEMALE)	0.029	-0.398	0.365	0.938	
**Random effects**					
Juvenile identity	0.077	0.004	0.299		
Residual	0.837	0.689	0.974		
**Repeatability**					
$R_{ADJ}|age$	0.123	0.006	0.272		

	Sociability N_{obs} = 108, N_{juv} = 27, N_{mom} = 27				
**Fixed effects**					
Intercept	14.822	12.358	16.722	<0.001	*
Age	-1.024	-1.731	-0.492	<0.001	*
Batch (ref = 1)	-0.142	-1.336	1.079	0.822	
Cohort (ref = 2014)	0.089	-2.231	2.299	0.976	
Body temperature	0.302	-0.378	0.753	0.534	
Sex (ref = FEMALE)	0.459	-1.969	2.411	0.850	
**Random effects**					
Juvenile identity	3.258	0.623	10.263		
Residual	7.377	5.717	11.268		
**Repeatability**					
$R_{ADJ}|age$	0.284	0.099	0.591		

	Aggression N_{obs} = 132, N_{juv} = 22, N_{mom} = 22			
**Fixed effects**				
Intercept	0.090	-0.440	0.437	0.922
Age	0.133	-0.117	0.290	0.368
Batch (ref = 1)	-0.301	-0.727	0.165	0.216
Cohort (ref = 2014)	0.210	-0.247	0.653	0.382
Body temperature	0.015	-0.168	0.232	0.640
Sex (ref = FEMALE)	-0.014	-0.540	0.353	0.694
**Random effects**				
Juvenile identity	0.008	0.001	0.340	
Residual	0.982	0.676	1.209	
**Repeatability**				
$R_{ADJ}|age$	0.008	0.001	0.269	

Table S4. Outcomes of linear mixed-effects models that only examined data for dominant skinks used for estimating repeatability (adapted R|time from Biro and Stamps [13]) of behavioural traits. These models also include the fixed factors of age, batch (1 or 2), cohort (2014 and 2015), body temperature, and sex (male or female), as well as the random effects of juvenile and mother identity. All continuous variables were mean centred. We highlighted important variables in each model with an asterix (*) to the right of the 95% credibility intervals and the *pMCMC* values.

	Exploration					Boldness					Sociability					Aggression				
	N_{obs} = 144, N_{juv} = 18, N_{mom} = 15					N_{obs} = 180, N_{juv} = 15, N_{mom} = 12					N_{obs} = 72, N_{juv} = 18, N_{mom} = 15					N_{obs} = 96, N_{juv} = 15, N_{mom} = 14				
**Fixed effects**	***β***	**2.5%**	**97.5%**	***pMCMC***																
Intercept	-0.041	-0.552	0.365	0.706		4.864	4.044	5.396	<0.001	*	16.617	13.798	20.844	<0.001	*	-0.314	-0.886	0.119	0.164	
Age	0.124	-0.055	0.334	0.142		-0.273	-0.416	-0.090	0.006	*	-0.918	-1.544	0.403	0.150		0.015	-0.259	0.154	0.844	
Batch (ref = 1)	0.425	0.117	0.773	0.016	*	-0.170	-0.496	0.087	0.176		-1.055	-2.705	0.352	0.104		-0.163	-0.576	0.244	0.366	
Cohort (ref = 2014)	-0.623	-1.034	-0.116	0.020	*	1.049	0.578	1.841	<0.001	*	-2.945	-6.165	0.835	0.150		0.758	0.150	1.232	0.012	*
Body temperature	0.261	0.115	0.450	<0.001	*	-0.204	-0.333	-0.029	0.032	*	0.560	-0.353	1.271	0.212		-0.030	-0.246	0.161	0.748	
Sex (ref = FEMALE)	0.712	0.005	0.998	0.036	*	0.174	-0.321	0.602	0.446		1.674	-2.208	3.856	0.466		0.263	-0.186	0.737	0.318	
**Random effects**	***$σ^2$***	**2.5%**	**97.5%**																	
Juvenile identity	0.026	0.002	0.700			0.011	0.001	0.482			8.895	0.116	39.688			0.007	0.001	0.345		
Mother identity	0.003	0.000	0.170			0.003	0.000	0.270			0.059	0.000	14.588			0.003	0.000	0.276		
Residual	0.835	0.636	1.106			0.816	0.669	1.032			8.921	4.885	11.985			0.704	0.476	0.914		
**Repeatability**	***R***	**2.5%**	**97.5%**																	
R_{ADJ}|*age*	0.161	0.002	0.445			0.009	0.001	0.365			0.622	0.189	0.886			0.007	0.001	0.345		

Table S5. Outcomes of linear mixed-effects models that only examined data for subordinate skinks used for estimating repeatability (adapted *R*|time from Biro and Stamps [13]) of behavioural traits. These models also include the fixed factors of age, batch (1 or 2), cohort (2014 and 2015), and body temperature, as well as the random effects of juvenile and mother identity. All continuous variables were mean centred. We highlighted important variables in each model with an asterix (*), and marginally important variables with a tilde (~) to the right of the 95% credibility intervals and the *pMCMC* values.

	Exploration N_{obs} = 128, N_{juv} = 16, N_{mom} = 14					Boldness N_{obs} = 180, N_{juv} = 15, N_{mom} = 14					Sociability N_{obs} = 64, N_{juv} = 16, N_{mom} = 14					Aggression N_{obs} = 72, N_{juv} = 12, N_{mom} = 11				
**Fixed effects**	***β***	**2.5%**	**97.5%**	***pMCMC***																
Intercept	0.442	0.095	0.859	0.028	*	5.226	4.574	5.640	<0.001	*	16.627	14.223	18.318	<0.001	*	-0.492	-0.974	0.144	0.146	
Age	0.286	0.079	0.493	0.016	*	0.042	-0.161	0.179	0.976		-0.777	-1.739	-0.058	0.038	*	-0.240	-0.564	-0.043	0.040	*
Batch (ref = 1)	-0.098	-0.497	0.284	0.590		-0.014	-0.327	0.267	0.878		-0.658	-2.114	1.138	0.588		0.098	-0.572	0.423	0.738	
Cohort (ref = 2014)	-0.567	-0.965	-0.054	0.018	*	0.762	0.200	1.448	0.016	*	-0.456	-2.282	2.907	0.842		0.577	0.047	1.354	0.034	*
Body temperature	0.136	-0.046	0.307	0.168		-0.100	-0.227	0.018	0.094	~	0.044	-0.733	0.689	0.974						
																0.224	0.045	0.516	0.040	*
**Random effects**	***σ*²**	**2.5%**	**97.5%**																	
Juvenile identity	0.011	0.001	0.211			0.238	0.002	0.888			0.049	0.001	10.869			0.011	0.001	0.642		
Mother identity	0.002	0.000	0.108			0.003	0.000	0.343			-0.010	0.000	8.010			0.002	0.000	0.382		
Residual	1.066	0.815	1.386			0.781	0.606	0.972			7.403	4.929	11.926			0.821	0.556	1.166		
**Repeatability**	***R***	**2.5%**	**97.5%**																	
R_{ADJ}|*age*	0.004	0.001	0.161			0.207	0.001	0.506			0.003	0.000	0.581			0.007	0.001	0.436		

Supplemental Figures

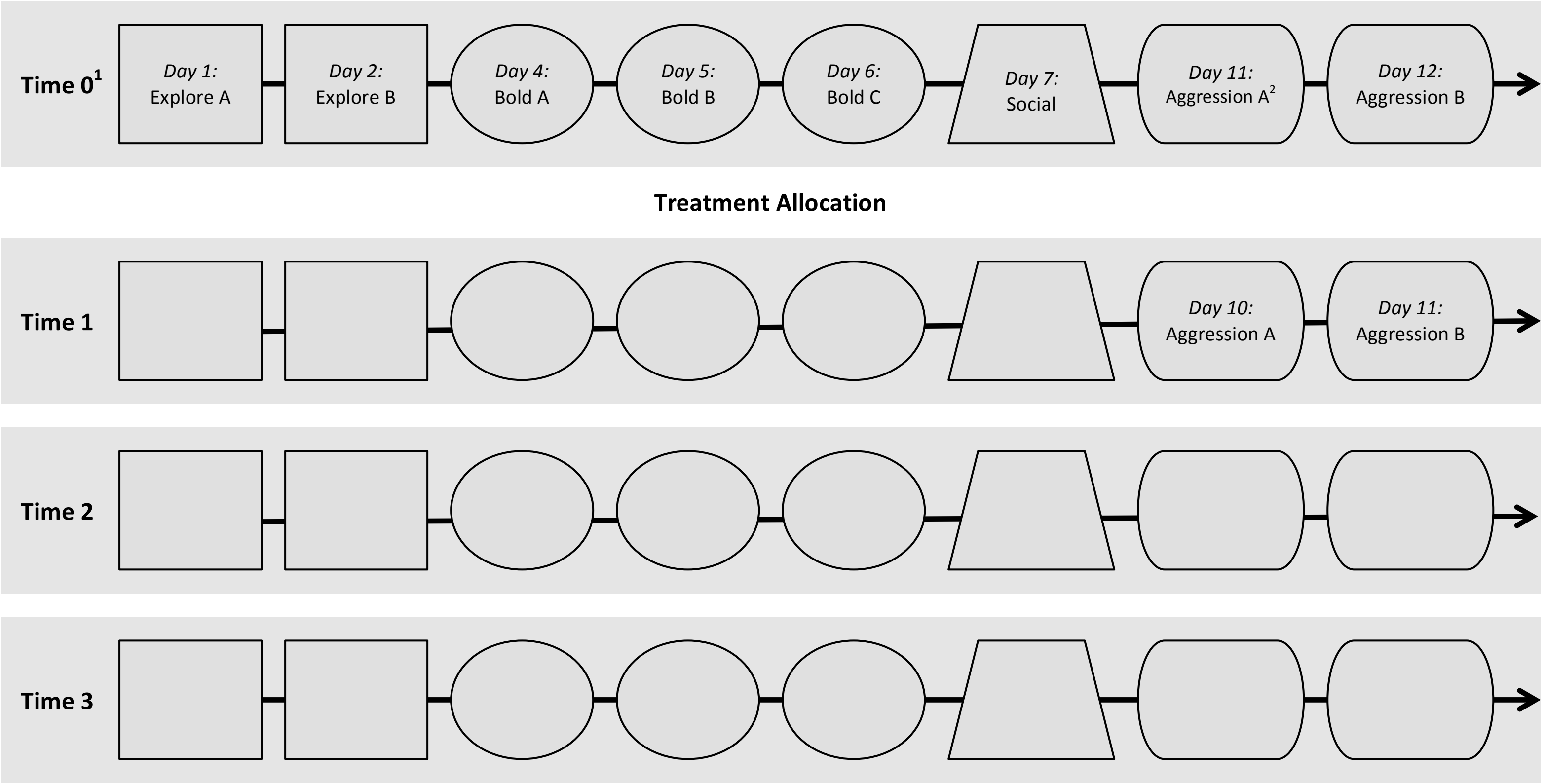

[1]Within each time period, the tree skinks were randomly separated into two experimental batches due to space constraints within our experimental room.
[2]Tree skinks from the 2014 cohort did not undergo the assays: Aggression A, and Aggression B.

Figure S1. Sequence of behavioural trait assays the 2014 (N = 28) and 2015 (N = 38) cohorts of tree skinks underwent to categorize their personality traits. The type of behavioural trait assay is represented with a different *shape*, and sequence is noted within the shape. At each time period (Time 0, 1, 2, and 3), we first categorized their exploratory behaviour with two repeated assays (represented by *rectangles*), boldness with three repeated assays (*circles*), sociability with one assay (*quadrilateral*), and aggression with two repeated assays (*rectangle with rounded ends*). The timing of aggression assays changed between Time 0 and Time 1, 2, and 3: from day 11 and 12, to day 10 and 11.

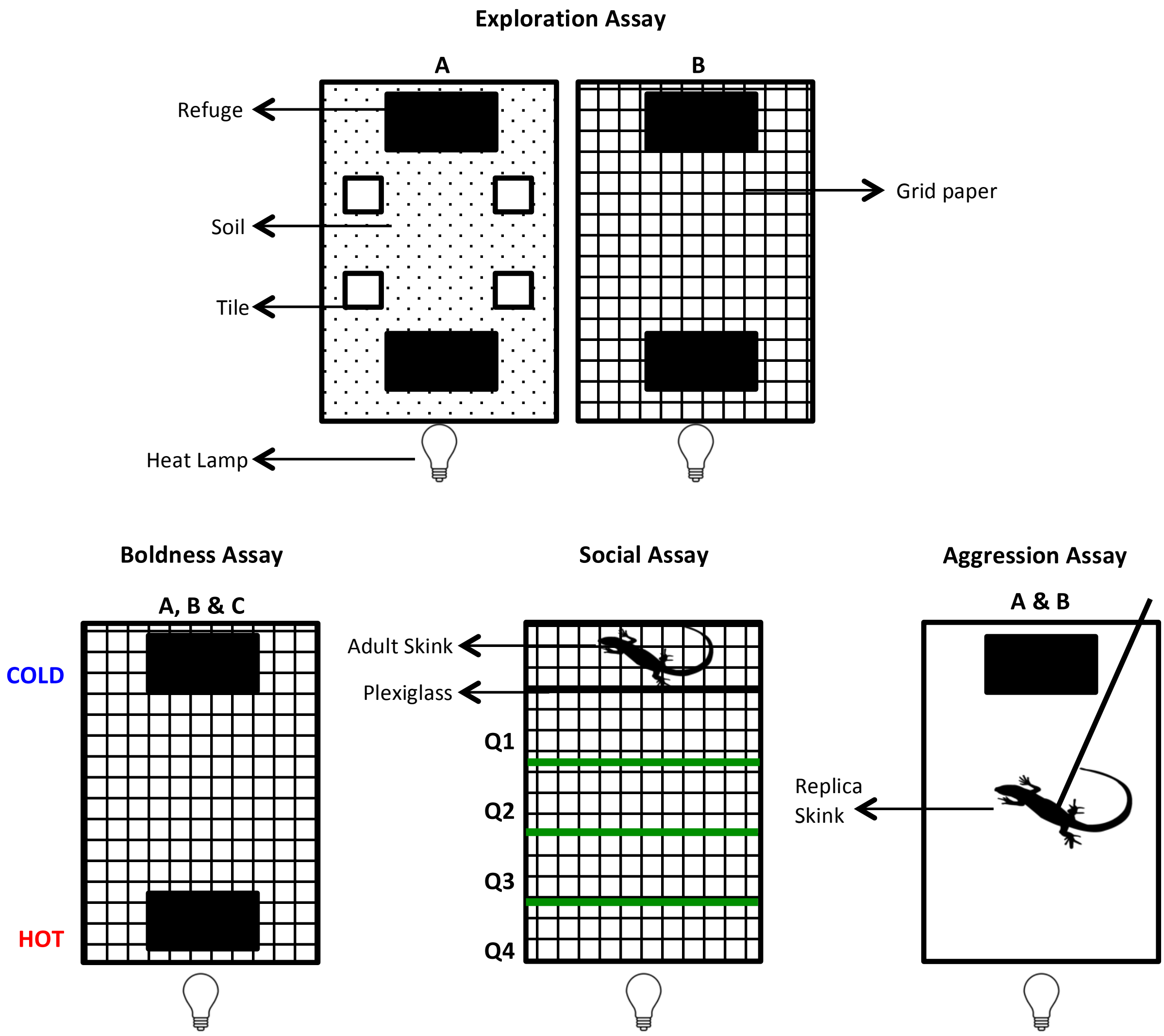

Figure S2. Experimental arena set-ups for our behavioural trait assays.

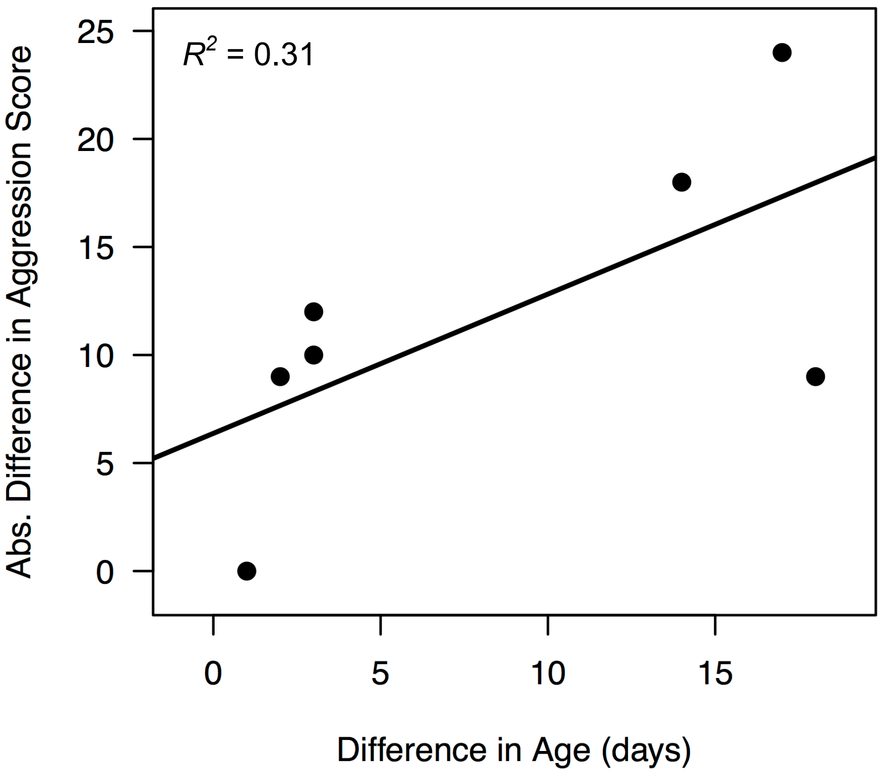

Figure S3. Socially-reared skink pairs ($N = 7$) that were more different in age (e.g., one individual born early in the season, and the other born late in the season) tended to also have a larger difference in aggression score (e.g., one very aggressive skink, and one very submissive skink). However, probably because of our small sample size, this relationship was not significant ($t_{1,\,5} = 1.914$, $P = 0.114$, $R^2 = 0.308$).
